# Anti-Inflammatory Diet Prevents Subclinical Colonic Inflammation and Alters Metabolomic Profile of Ulcerative Colitis Patients in Clinical Remission

**DOI:** 10.3390/nu14163294

**Published:** 2022-08-11

**Authors:** Ammar Hassanzadeh Keshteli, Rosica Valcheva, Cheryl Nickurak, Heekuk Park, Rupasri Mandal, Kendall van Diepen, Karen I. Kroeker, Sander Veldhuyzen van Zanten, Brendan Halloran, David S. Wishart, Karen L. Madsen, Levinus A. Dieleman

**Affiliations:** 1 Department of Medicine, University of Alberta, Edmonton, AB T6G 2X8, Canada; 2 Department of Biological Sciences, University of Alberta, Edmonton, AB T6G 2E9, Canada; 3 Alberta Health Services, Edmonton, AB T6G 2E9, Canada; 4 Department of Computing Science, University of Alberta, Edmonton, AB T6G 2X8, Canada; 5 Division of Gastroenterology, University of Alberta, 130 University Campus, 2-24 Zeidler Ledcor Building, Edmonton, AB T6G 2X8, Canada

**Keywords:** ulcerative colitis, diet, clinical trial

## Abstract

A relationship between ulcerative colitis (UC) and diet has been shown in epidemiological and experimental studies. In a 6-month, open-label, randomized, placebo-controlled trial, adult UC patients in clinical remission were randomized to either an “Anti-inflammatory Diet (AID)” or “Canada’s Food Guide (CFG)”. Menu plans in the AID were designed to increase the dietary intake of dietary fiber, probiotics, antioxidants, and omega-3 fatty acids and to decrease the intake of red meat, processed meat, and added sugar. Stool was collected for fecal calprotectin (FCP) and microbial analysis. Metabolomic analysis was performed on urine, serum, and stool samples at the baseline and study endpoint. In this study, 53 patients were randomized. Five (19.2%) patients in the AID and 8 (29.6%) patients in the CFG experienced a clinical relapse. The subclinical response to the intervention (defined as FCP < 150 µg/g at the endpoint) was significantly higher in the AID group (69.2 vs. 37.0%, *p* = 0.02). The patients in the AID group had an increased intake of zinc, phosphorus, selenium, yogurt, and seafood versus the control group. Adherence to the AID was associated with significant changes in the metabolome, with decreased fecal acetone and xanthine levels along with increased fecal taurine and urinary carnosine and p-hydroxybenzoic acid levels. The AID subjects also had increases in fecal Bifidobacteriaceae, Lachnospiraceae, and Ruminococcaceae. In this study, we found thatdietary modifications involving the increased intake of anti-inflammatory foods combined with a decreased intake of pro-inflammatory foods were associated with metabolic and microbial changes in UC patients in clinical remission and were effective in preventing subclinical inflammation.

## 1. Introduction

Ulcerative colitis (UC) is a subtype of inflammatory bowel disease (IBD) characterized by chronic relapsing and remitting inflammation of the colonic mucosa [1]. Diarrhea and the presence of blood in the stool are the most common symptoms of active UC. UC prevalence and incidence have been increasing worldwide [2]. Although the exact pathophysiological mechanisms of UC development remain unknown, it has been suggested that a combination of several factors, including genetic predisposition, epithelial barrier defects, dysregulated immune responses, microbial dysbiosis, and environmental factors, plays a major role [1,2].

As shown in several experimental and epidemiological studies, dietary factors are among the potential environmental contributors to UC development. Studies have linked the high intake of soft drinks and sucrose and *n*-6 polyunsaturated fatty acids (PUFAs) and the low intake of fruits, vegetables, and *n*-3 PUFAs with an association with UC [3,4,5,6,7]. Although the exact mechanisms responsible for the association between diet and UC are unknown, several mechanisms have been suggested. An unhealthy dietary pattern such as a Western diet has been linked to dysbiosis of the gut microbiome, epithelial barrier dysfunction, and persistent pro-inflammatory mucosal immune responses that may ultimately trigger and perpetuate increased chronic colonic inflammation [4].

Multiple disease relapses result in impaired quality of life in IBD patients [8] and an increased risk of colitis-associated colorectal cancer in patients with longstanding UC and Crohn’s colitis [9]. Many UC patients attribute their disease relapses to diet, and there are several, mostly observational and retrospective, studies of dietary factors that have been associated with the increased risk of UC relapse [10,11]. However, according to a recent Cochrane systematic review, a consensus on the composition of evidence-based dietary interventions in UC patients is required, and there is a need for more high-quality, well-powered, randomized, controlled trials to assess the efficacy of dietary interventions [12]. In the present randomized controlled pilot trial, we aimed to assess whether a diet based on dietary components with proven anti-inflammatory properties would be effective in maintaining remission in adult UC patients and to determine the potential protective mechanisms. 

## 2. Materials and Methods

### 2.1. Study Design and Patients

This study was an open-label, randomized, controlled, parallel-group study conducted at the University of Alberta in Edmonton, Alberta, Canada, from 2014 to 2017 on UC patients aged 18 to 75 years. The inclusion criteria included patients who were in clinical remission (partial Mayo score of ≤2, with a rectal bleeding subscore of ≤1)) [13] but who also had a documented history of a UC clinical relapse (partial Mayo of score > 2) in the previous 18 months. Participants could be on any standard UC medications if they were on a stable dosage of oral 5-aminosalicylic acid (5-ASA) for at least 2 weeks and on a stable dosage of immunosuppressants or anti-tumor necrosis factor (TNF)-α for at least 2 months. We excluded patients who were on corticosteroids or antibiotics within 2 weeks of enrollment. In addition, the study subjects were excluded if they were pregnant or lactating, had significant co-morbidities, or a history of colectomy. All participants needed to be able to communicate in English.

After stratification for sex and the use of anti-TNF medications, the subjects were randomized 1:1 into the Anti-inflammatory Diet (AID) or the control group using the randomization module of REDCap (Research Electronic Data Capture) [14]. The study protocol was approved by the Health Research Ethics Board, University of Alberta (Pro00035413), and written informed consent was obtained from all the participants (ClinicalTrials.gov (accessed on 10 June 2021). Identifier: NCT02093780). Neither the patients nor the public were involved in the design, or conduct, or reporting, or dissemination plans of our research.

### 2.2. Intervention

The patients who were randomized to the control arm received dietary recommendations (not menu plans) to comply with Canada’s Food Guide (CFG), version 2007 [15], with respect to daily recommended food group servings (Supplementary Table S1). The CFG was primarily developed to help Canadians achieve a healthy, balanced diet and to reduce the risk of obesity, type 2 diabetes, heart disease, certain types of cancer, and osteoporosis. The patients in the CFG group had the same amount of face-to face counselling at the baseline, at months 1, 3, and 6, or at relapse and telephone visits by the same dietitian at months 2, 4, and 5.

The participants randomized to the AID group were provided with 45 to 60 min face-to-face dietary counselling by the registered dietitian at the baseline, at months 1, 3, and 6, or at relapse. At months 2, 4, and 5, the dietary recommendations were delivered by the same dietitian through 30 min telephone counselling sessions. They were instructed to follow a structured four-week menu plan that included recipes and nutrition tips. The menu plan was a modified version of the previously developed menu plan for the management and prevention of type 2 diabetes, which has been described elsewhere [16]. The original anti-diabetic menu plan was modified to emphasize the specific foods shown in the literature to improve IBD-related symptoms or prevent IBD relapses. In terms of the number of food group servings, the menus followed the recommended food group servings outlined in the CFG [15]. On average, they provided 2000 kcal with 54%, 19%, and 27% of the energy from carbohydrates, protein, and fat, respectively. Each recipe included ways to increase or reduce caloric intake by 200 kcal. Menu plans and nutrition tips for the AID group were designed to increase the participants’ intake of antioxidants, dietary fibers, probiotics, and *n*-3 PUFA and decrease the consumption of red meat, processed meat, and added sugar. For example, foods high in antioxidants, such as berries, were incorporated into the daily menu plan. Other foods high in antioxidants, such as legumes or pulses, were also incorporated into several days of each of the four weeks of menus. Probiotics from foods, such as plain yogurt with active culture or the amount of probiotics noted on the product label, were included. In addition, a significant number of recipes included foods with high prebiotic/dietary fiber content, such as onion, garlic, and asparagus. The menu plan also ensured that the participants were consuming two servings of fish weekly. Furthermore, at least 50% of the weekly fish recipes involved consuming *n*-3 PUFA enriched fish, such as salmon. To facilitate adherence to the menus, several recipes, cooking tips, weekly grocery lists and a list of Alberta-produced foods and places to obtain them were also provided to the participants. In addition, the participants randomized to the AID group were provided with a food list from which they could choose their preferred food items. This could help the dietitian direct an individualized dietary plan for the participants to incorporate their daily dietary requirements. 

### 2.3. Demographic and Clinical Assessments

Demographic and clinical information was collected at the baseline. At the baseline and at the end of the study, weight and height were measured, and body mass index (BMI) was calculated. We used the Short Inflammatory Bowel Disease Questionnaire (SIBDQ) [17] to assess the health-related quality of life in all the patients at the baseline and at month 6 or at clinical relapse. Disease activity was assessed at the baseline and then monthly, using a partial Mayo score that included stool frequency, rectal bleeding, and a physician’s assessment, with total values ranging from 0 to 9 [13]. 

### 2.4. Dietary Assessments 

To assess adherence to the dietary recommendations and changes in the dietary intake from the baseline to the end of the trial, monthly self-administered 24 h dietary recalls were used. The dietary intake data for 24 h dietary recalls were collected and analyzed using the Automated Self-Administered 24 h (ASA24) Dietary Assessment Tool 2014, developed by the National Cancer Institute, Bethesda, MD [18]. A validated dietary inflammatory index (DII) score, which assesses the inflammatory potential of a diet, was calculated using the method proposed by Shivappa et al. [19]. A higher DII score (more positive values) indicates a more inflammatory diet, and a lower DII score (more negative values) indicates a less inflammatory diet.

### 2.5. Laboratory Assessments

Blood, urine, and stool samples were collected from all the patients during the clinic visits (baseline, months 1, 3 and 6). Fecal calprotectin (FCP) was measured in the stool samples at the baseline and at months 1, 3, and 6 (or at the time of relapse) using an enzyme-linked immunosorbent assay with monoclonal antibodies specific to calprotectin (Bühlmann Laboratories AG, Basel, Switzerland). The subclinical response to the intervention was defined as FCP < 150 µg/g at the endpoint. 

Targeted metabolomic assays that measure up to 160 metabolites each, including amino acids, organic acids, short-chain fatty acids, sugars, lipids, acylcarnitines, and biogenic amines, were conducted on the urine, serum, and stool samples at the baseline and at the end of the trial (month 6 or at relapse). The urine samples were assayed using a combined direct infusion (DI-)/liquid chromatography (LC-) tandem mass spectrometry (MS/MS) as well as a gas-chromatography (GC-) MS assay. DI- LC MS/MS and nuclear magnetic resonance (NMR) spectroscopy were used to identify and quantify the metabolites in the serum samples. NMR was used to identify and quantify metabolites in the stool samples. All the metabolomic assays were conducted at The Metabolomics Innovation Center-TMIC (Edmonton, AB, Canada) following previously described protocols [20,21,22,23]. 

Genomic DNA was extracted from stool samples using the FastDNA Spin Kit for feces (MP Biomedicals, Lachine, QC, Canada) and quantified using the PicoGreen DNA quantification kit (Invitrogen, Carlsbad, CA, USA). Fecal microbial composition was assessed using Illumina’s established 16S rRNA amplicon sequencing method and the MiSeq DNA sequencing platform. No deviations from the manufacturer’s protocol were made. A segment of the V3 and V4 region of the 16S gene was amplified with gene-specific primers (aligning to 341 and 805 bp in the gene) that also included an adapter sequence overhang: Bact_16s_ILL1_341mF 5′-TCG TCG GCA GCG TCA GAT GTG TAT AAG AGA CAG CCT ACG GGN GGC WGC AG-3′, Bact_16s_ILL1_805mR 5′- GTC TCG TGG GCT CGG AGA TGT GTA TAA GAG ACA GGA CTA CHV GGG TAT CTA ATC C-3′. This PCR reaction was cycled 25 times, and the resulting reaction was purified using bead-based clean-up followed by an 8-cycle PCR reaction using Illumina’s proprietary bar-coding primers that also align to the adapter sequence. After a second clean-up, the bar-coded libraries were diluted, denatured, pooled, and run using a V3 300 bp reagent cartridge on the MiSeq system. The Divisive Amplicon Denoising Algorithm version 2 (DADA2 1.12.1) was used for quality filtering, trimming, error correction, exact sequence inference, chimera removal, and the generation of amplicon sequence variant (ASV) tables [24]. Taxonomic classification was performed using a Naïve Bayes classifier trained using the GreenGenes 97% clustered sequences (version 13_8), obtained from https://benjjneb.github.io/dada2/training.html (accessed on 15 July 2021)

### 2.6. Study Outcomes

The primary outcome was the clinical relapse of UC, defined as a partial Mayo score of > 2, measured at each face-to-face or telephone visit. The secondary outcomes were changes in FCP, the subclinical response rate (FCP < 150 µg/g at the endpoint), the health-related quality of life scores, and the metabolomic and gut microbial profiles from the baseline to the end of the trial.

### 2.7. Sample Size Calculation

With a power of 80%, a type I error of 5%, a clinical relapse rate of 10% in the AID group and 40% in the control group, and a drop-out rate of 10%—a total sample size of 70 participants was calculated to be required in this study (35 participants in each group).

### 2.8. Statistical Analysis

An intention-to-treat approach was used to analyze the data, such that the data from all the patients were analyzed according to the diet to which they were randomized. The Kolmogorov–Smirnov test and histograms were used to assess the normality of the data distribution. Continuous and categorical variables are presented as mean ± SD or median (interquartile range) and number (%), respectively. Logarithmic transformation was used to normalize those data that were not normally distributed. Chi-square or Fisher’s exact tests were used to compare qualitative variables between groups. A Student’s *t*-test or a Mann–Whitney U test was used to compare quantitative variables between groups, where appropriate. The baseline values and endpoint measures were compared within each group using a paired *t*-test or a Wilcoxon signed-rank test. The FCP changes from the baseline to months 1, 3, and 6 within each group were assessed using analysis of variance with the Friedman test (a nonparametric test). To examine the effect of intervention on fecal calprotectin, split-plot repeated measures ANOVA (split-plot rANOVA) was used, in which the effect of time, intervention (effects between groups), and time × intervention interactions were assessed. For this analysis, we controlled for the baseline levels of the outcome variables and the potential confounding variables. SPSS version 20.0 (IBM, Armonk, NY, USA) was used for statistical analysis, and *p* < 0.05 was considered statistically significant.

For the metabolomic analysis, the metabolites with at least 50% missing values were removed from further analysis. For those analytes with < 50% missing values, the missing values were replaced by half of the minimum positive values in the original dataset. Concentrations of urinary metabolites (μmol/L) were normalized to creatinine (mmol/L) and reported as the ratio (μmol/mmol). Concentrations of fecal metabolites were normalized to the dry weight of the stool sample and reported as μmol/gr. Multivariate statistical analysis was performed using partial least squares discriminant analysis (PLS-DA). Permutation analysis using random resampling (*n* = 2000) of the two groups of patients (i.e., AID baseline vs. CFG baseline, AID baseline vs. end of trial, and CFG baseline vs. end of trial) was conducted, and a *p* value was determined. Variable importance in projection (VIP) scores were used to identify the major metabolites responsible for the discrimination between the metabolomic profiles of the two groups of patients. The VIP score indicates the contribution of each feature to the regression model. Higher values of VIP scores indicate a greater contribution of the metabolites to the group separation. The MetaboAnalyst 4.0 [25] was used for all metabolomic-associated statistical analyses.

For microbial analysis, the ASV tables were imported into R 3.6.1 to calculate α-diversity and β-diversity metrics, using the Phyloseq v1.28.0 package [26]. Based on α-diversity rarefaction, the samples were included in the analyses if the rarefaction curves reached a plateau and a minimum cut-off of 10,000 counts was exceeded. Differential abundance analysis for bacterial ASVs was performed using DESeq2. β-diversity was analyzed using permutational multivariate analysis of variance. Analyses were adjusted for age, gender, and fecal calprotectin levels at the baseline. All P values were adjusted by the Benjamini–Hochberg method to control the false discovery rate at 5%. 

To investigate and visualize the interactions between changes in DII, metabolites, gut microbial composition, and FCP levels from the baseline to the last visit, the correlations between these features were calculated and visualized. For this purpose, the debiased sparse partial correlation (DSPC) algorithm option of the Metscape v3.1.321 [27], which is a plug-in for Cytoscape [28], was applied. The results were visualized as weighted networks where nodes represented different features and edges represented partial correlation coefficients. 

## 3. Results

### 3.1. Demographics: AID vs. CFG 

**Figure 1:**
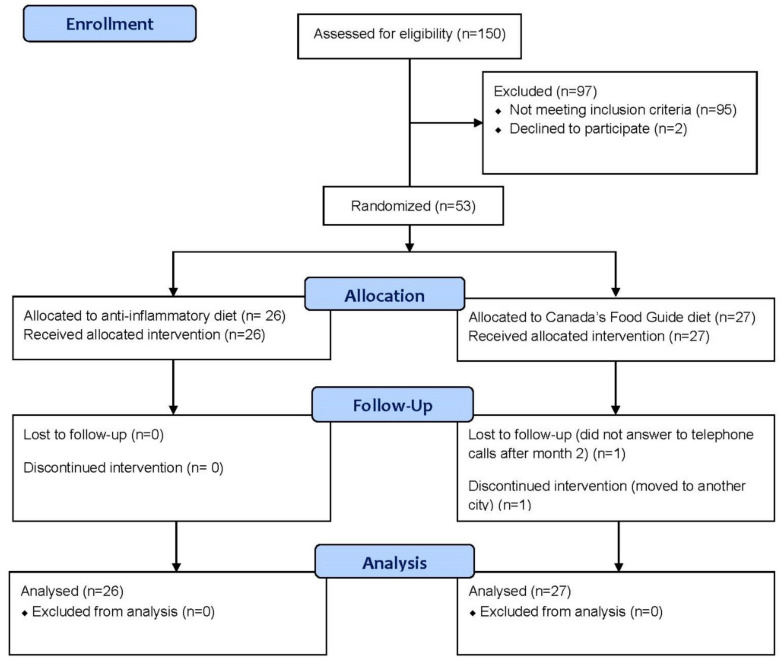
CONSORT flow diagram.

Fifty-three UC patients in clinical remission were randomized to the two dietary intervention groups: 26 to the AID and 27 to the CFG group. Due to the feasibility issues (being a single-center study, the eligibility criteria, etc.) the pre-planned number of participants could not be recruited into the study. The flow of participants through the trial is presented in Figure 1. Two patients left the study just before the clinic visit at month 3; however, their data were used for statistical analysis by following the last-observation-carried-forward method. The mean age of the participants was 41.4 ± 14.7 years; 34 (64.2%) participants were female; 25 (47.2%) had pan-colitis; 22 (41.5%) had left-sided colitis; 16 (30.2%) were on immunosuppressants; and 13 (24.5%) were on anti-TNF medications. The baseline demographic and clinical characteristics of the participants across the two diet groups are summarized in Table 1. There were no significant differences in the baseline characteristics between the groups.

### 3.2. Clinical Relapse and Changes in Quality of Life: AID vs. CFG

In total, 13 (24.5%) patients experienced a clinical relapse during the intervention. Five (19.2%) patients in the AID group and eight (29.6%) patients in the CFG diet group had a UC clinical relapse, which was not statistically significant (*p* = 0.38). The SIBDQ scores (to assess quality of life) did not change significantly from the baseline to the last visit, either in the control group (5.5 ± 0.7 vs. 5.5 ± 0.9, *p* = 0.80) or in the AID group (5.5 ± 0.9 vs. 5.6 ± 0.8, *p* = 0.56).

### 3.3. Changes in FCP Levels: AID vs. CFG

**Figure 2:**
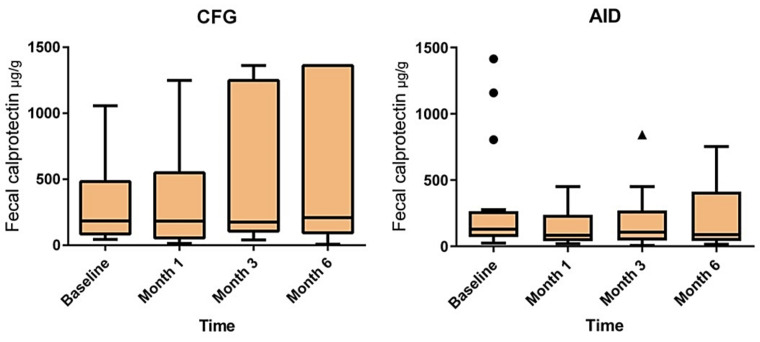
Changes in fecal calprotectin levels from baseline to the end of the trial. While there was a statistically significant increase in fecal calprotectin from baseline to month 6 or at time of relapse in patients randomized to the Canada’s Food Guide (CFG) diet (*p* = 0.002), patients in the Anti-Inflammatory Diet (AID) showed a slight decrease in their fecal calprotectin levels during the same period (*p* = 0.053). Each box shows the median and interquartile range values, and a Friedman test was used to compare FCP median values from baseline to the last visit in each group. Changes in FCP from baseline to the last visit between the two groups after adjusting for baseline FCP values was also statistically significant (*p* = 0.02) using split-plot repeated measures ANOVA.

The baseline FCP values did not differ significantly between the two groups (Table 1). The changes in FCP from the baseline to month 6 or the time of relapse in the two diet groups are presented in Figure 2. While there was a trend towards a decrease in FCP from the baseline to the end of the trial in the patients randomized to the AID group (*p* = 0.053), the FCP values increased significantly in patients randomized to the CFG group from the baseline to month 6 (*p* = 0.002). In addition, the comparison of these FCP changes between the two diet groups using split-plot rANOVA (adjusting for the baseline FCP in each group) was statistically significant (*p* = 0.02). Furthermore, the subclinical response to the dietary intervention, defined as FCP < 150 µg/g at the endpoint, was significantly higher in the AID group in comparison to the CFG group (69.2 vs. 37.0%, *p* = 0.02).

### 3.4. Changes in Dietary Intake: AID vs. CFG

**Figure 3:**
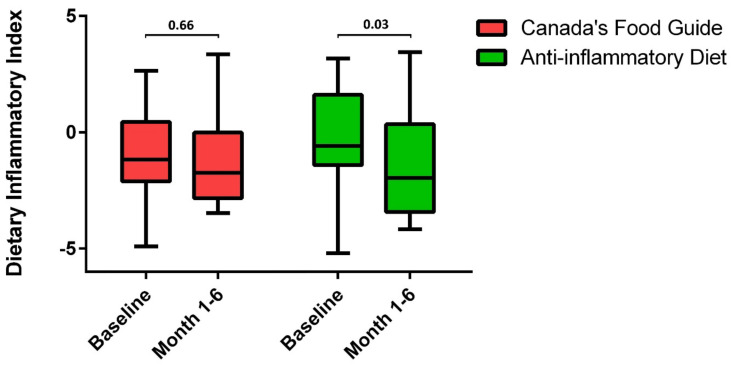
Comparison of dietary inflammatory index (DII) scores (median and interquartile range) from baseline to the end of the trial between the two intervention groups. There was a significant decrease in DII scores in patients randomized to the Anti-inflammatory Diet.

While there were no statistically significant changes (relative to the baseline) in the dietary intake of the patients randomized to the CFG group, the patients in the AID group significantly increased their intake of fiber, zinc, phosphorus, selenium, yogurt, and seafood (Supplementary Table S1). The DII scores at the baseline were not significantly different between the two diet groups (−1.4 (−0.6–1.6) in AID vs. −2.1 (−1.2–0.5) in CFG, *p* = 0.29). While there was a significant decrease in the total DII score of the patients in the AIG group, the patients in the CFG group did not experience any significant changes in their DII (Figure 3). Increases in FCP were significantly correlated with decreases in the intake of yogurt (r_s_ = −0.39, *p* = 0.01), poultry (r_s_ = −0.34, *p* = 0.01), seafood (r_s_ = −0.29, *p* = 0.05). Increases in FCP were also significantly correlated to increases in the intake of fruit juices (r_s_ = 0.38, *p* = 0.01), cured meat (r_s_ = 0.29, *p* = 0.04), and saturated fatty acids (r_s_ = 0.28, *p* = 0.05), from the baseline to the end of the trial. 

### 3.5. Changes in Gut Bacterial Composition: AID vs. CFG

**Figure 4:**
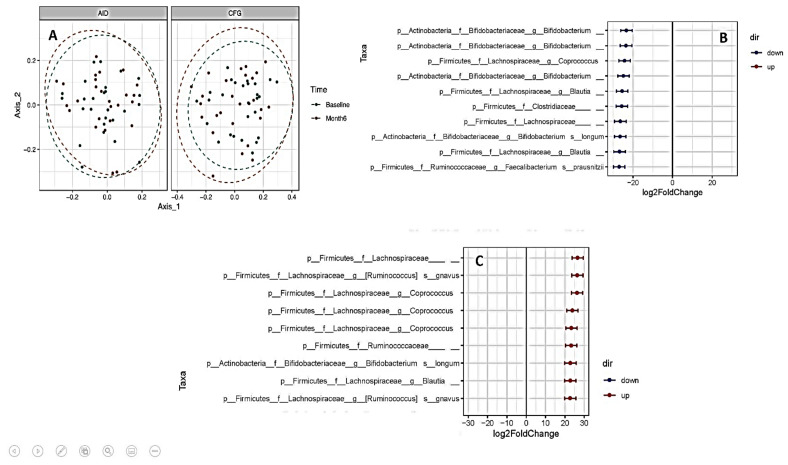
(**A**) Principal Coordinates Analysis (PCoA) plot of beta-diversity for bray distance matrix showing no significant changes in gut microbial composition in the Anti-inflammatory Diet (AID) and Canada’s Food Guide (CFG) groups from baseline to month 6 or time of clinical relapse. (**B**) Differential abundance testing showing significant changes in several bacterial amplicon sequence variants (ASVs) from baseline to the end of the intervention in the Canada’s Food Guide diet group (adjusted *p* < 0.01, absolute fold change >5). (**C**) Differential abundance testing showing significant changes in several bacterial amplicon ASVs from baseline to the end of the intervention in the Anti-Inflammatory Diet group (adjusted *p* < 0.01, absolute fold change >5).

A microbial profile of stool samples from the patients in the two diet groups at the baseline and at the last visit (either month 6 or the time of relapse) is shown in Supplementary Figure S1. As presented in the Principal Coordinates Analysis (PCoA) plots (Figure 4A), there were no significant differences in the gut bacterial composition of patients in the two diet groups from the baseline to month 6 or at the time of relapse. The alpha diversity scores (Chao 1 estimator and Shannon index) also did not change significantly from the baseline to the end of the trial in the two intervention groups (Supplementary Figure S2). However, as shown in Figure 4B,C, several bacterial ASVs changed significantly from the baseline to month 6 or the time of relapse in the two intervention groups. While there was a significant decrease in *Bifidobacteriaceae, Lachnospiraceae*, *Clostridiaceae*, and *Ruminococcaceae* in the CFG group, there was a significant increase in the abundance of *Bifidobacteriaceae, Lachnospiraceae*, and *Ruminococcaceae* in the AID group from the baseline to the last visit. 

### 3.6. Changes in Metabolome: AID vs. CFG

**Figure 5:**
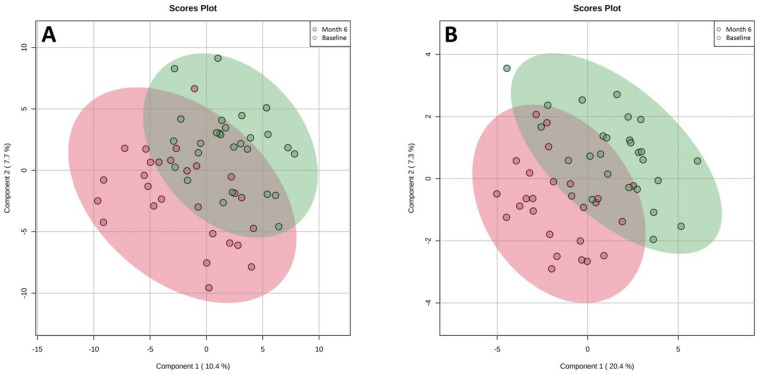
Partial least squares discriminant analysis plot comparing the metabolomic profiles of patients in the two diet groups from baseline to month 6 or time of relapse. While patients in the Canada’s Food Guide diet (**A**) did not have any significant changes in their metabolome (*p* = 0.93, R^2^ = 0.26, Q^2^ = −0.38), patients randomized to the Anti-Inflammatory Diet (**B**) group showed a significant change in their metabolomic profiles from baseline to the end of the intervention (*p* = 0.01, R^2^ = 0.74, Q^2^ = 0.27).

Using a combination of the LC-MS, GC-MS, and NMR metabolomic platforms, we could identify and quantify 184, 122, and 49 metabolites in the serum, urine, and stool samples, respectively. The metabolomic profiles of the patients between the two diet groups at the baseline were not significantly different from each other (*p* = 0.31). The comparison of the metabolome in the CFG patients did not show any significant changes from the baseline to month 6 or the time of relapse (Figure 5A). However, there was a significant separation of the metabolomic profiles of the patients in the AID group from the baseline to the end of the trial (Figure 5B). The VIP scores and concentrations of the major metabolites responsible for this separation are presented in Table 2. The AID intervention was associated with decreases in phosphatidylcholine acyl-alkyl (PC ae) C38:3 (urine), acetone (stool), and xanthine (stool) and increases in PC ae C38:5 (urine), pyruvic acid (serum), and taurine (stool).

### 3.7. Interaction between Changes in Diet, Metabolome, Microbiome, and FCP

**Figure 6:**
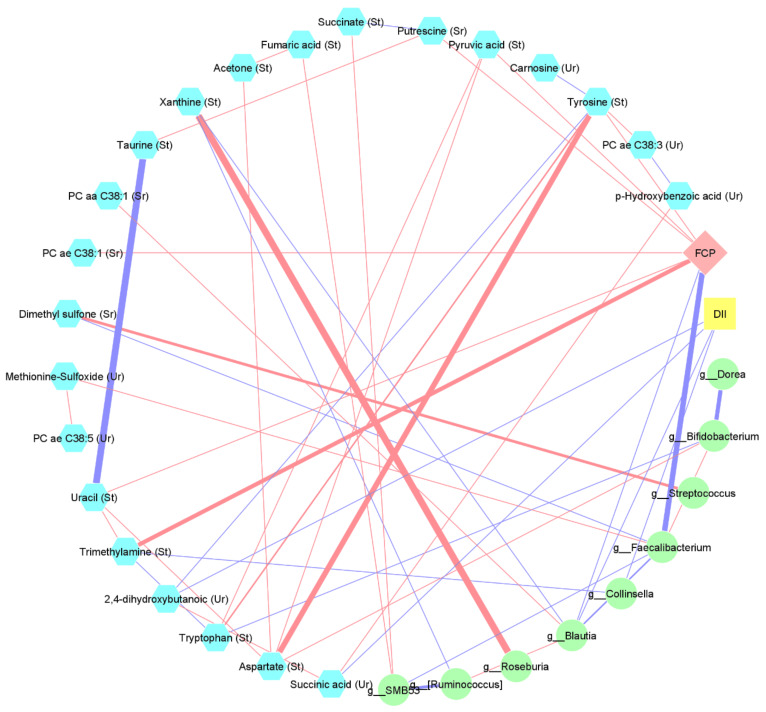
Correlation between changes in dietary inflammatory index (DII) score, fecal calprotectin (FCP), metabolites (urine: Ur, stool: St, and serum: r), and gut bacterial composition (genus level) from baseline to last visit (time of relapse or month 6). The statistically significant correlations were filtered using |Spearman’s rank correlation| >0.3, and subsequently, a correlation network was built. Only metabolites and bacteria that were correlated with either DII or FCP are shown. Blue lines represent negative correlations, and red lines represent positive correlations.

The correlation network between the changes in the metabolites levels, the bacterial relative abundances at the genus level, the DII (as inflammatory potential of diet), and the FCP levels from the baseline to the time of relapse or the month 6 visit are presented in Figure 6. Although the changes in the total DII scores were not directly correlated with the changes in FCP levels, the decreased DII scores (i.e., increased intake of anti-inflammatory foods) were correlated with significant changes in gut bacterial composition (increased *Collinsella* and *Blautia*) and metabolites levels (increased urinary succinic acid and 2,4-dihydroxybutanoic acid). As shown in Figure 6, the changes in these bacteria and metabolites were associated with changes in the levels of other metabolites and the relative abundancies of several bacteria which were correlated with changes in the FCP levels from the baseline to the last visit. 

## 4. Discussion

Our study is the first 6-month-dietary randomized controlled intervention study to assess prevention of relapses in UC. In this study, the patients were consuming their own choice of preferred foods within the principles of the diet intervention group and closely monitored by a dietitian for adherence. Although there were no significant differences in clinical relapse in this 6-month RCT, we found that adherence to the AID maintained and even showed a trend towards reduced FCP levels, suggesting that this diet may have a beneficial role in preventing the onset of subclinical colitis. Importantly, we also found significant changes in the serum, urine, and stool metabolomes and changes to specific fecal bacteria following the AID in comparison to the CFG dietary recommendations. 

The role of diet in the pathogenesis of IBD has been reported in several animal and epidemiological studies. It has been suggested that a Western diet characterized by a high content of refined carbohydrates, saturated fatty acids, red meat, and processed meat and a low content of fruits, vegetables, legumes, and fibers increases the risk of IBD development through significant pro-inflammatory impacts on the host immune system and microbial composition or function [4,29]. In addition, prospective cohort studies have reported several dietary factors, such as red meat intake and processed meat intake, to be associated with the increased risk of disease relapse in UC patients [30]. Many IBD patients also believe that dietary factors are responsible for their disease development or relapse of symptoms. According to a large cross-sectional study in the UK [31], about half of IBD patients believed that diet could be the initiating factor in IBD and/or could trigger a flare. In addition, Limdi et al. [31] reported that 66% of IBD patients deprived themselves of their favorite foods to prevent relapses and that such dietary restrictions may eventually lead to various nutritional deficiencies. However, there are very few evidence-based dietary recommendations for relapse prevention in IBD patients, partly due to a lack of well-designed, randomized dietary interventions. 

In the present study, we did not find a significant difference in clinical relapse rate between the two dietary interventions. It should be noted that our study was not sufficiently powered to detect a small statistically and clinically important difference in the clinical relapse rate between the AID and CFG diets. However, we found that the AID prevented a statistically significant increase in FCP, which is an important objective marker of subclinical colonic inflammation and a strong predictor of future disease relapse [32]. Therefore, a larger sample size and/or longer duration study might have resulted in a statistically significant difference in the clinical relapse rate between the two groups. However, our 6-month study is the longest duration dietary intervention study conducted on UC patients so far. 

The AID used in the present study was characterized by an increased intake of antioxidants, dietary fibers, probiotics, and *n*-3 PUFA and a decreased intake of red meat, processed meat, and added sugar. The AID design was based on our current understanding of the role of dietary factors in the pathogenesis or disease course in IBD patients. For instance, red and processed meat intake was shown to be related to an increased risk of UC relapse [30]. Furthermore, the consumption of fruits and vegetables as two major sources of antioxidants and dietary fiber was related to decreased odds of UC development in a meta-analysis [33]. Another recent meta-analysis study found a negative association between long-chain *n-3* PUFA intake and UC development [34]. 

The assessment of dietary patterns and indices has become more popular in nutrition research, considering that we eat complex combinations of foods, rather than individual nutrients and food groups. In the present study, in addition to comparing single nutrients or foods, we used DII scores to assess compliance and changes in the diet during the intervention. The DII was developed to characterize the inflammatory potential of a diet based on evidence from human studies, cell culture, and animal experiments [19]. Higher DII scores, which are reflective of a higher intake of inflammation-inducing foods or nutrients, have been related to several chronic conditions, such as cardiometabolic diseases, respiratory diseases, mental conditions, and cancers. In addition, a high DII was associated with increased odds of UC in a case-control study [35]. In the present study, we found a significant decrease in DII scores as a result of the AID intervention due to the increased intake of anti-inflammatory nutrients or foods and the decreased intake of pro-inflammatory foods or nutrients. The observed reduction in DII scores also confirms the participants’ compliance with the diet. Furthermore, we found that increased consumption of yogurt, seafood, or poultry and decreased consumption of cured meat or saturated fatty acids were correlated with decreased FCP levels from the baseline to the end of the study. This finding highlights the anti-inflammatory nature of yogurt, poultry, and seafood and the inflammatory nature of cured meat and saturated fatty acids. After assessing dietary intake using validated 24 h recalls, we found a significant increase in seafood intake, as a source of *n-3* PUFAs, in patients randomized to the AID group. Pre-clinical studies have identified that *n-3* PUFAs can affect the cell membrane composition and function, eicosanoid synthesis, and signaling, as well as the regulation of gene expression [36]. A recent meta-analysis of observational studies reported a negative association between fish consumption and the risk of Crohn’s disease (CD), as well as an inverse association between dietary long-chain n-3 PUFAs and the risk of UC development [34]. Furthermore, we detected that the patients in the AID group had a significant increase in zinc, phosphorus, and selenium intake. Deficiencies of zinc, phosphorus, and selenium have been found in IBD patients [37]. As lower levels of these micronutrients were associated with disease onset or exacerbation of the inflammation, some experimental studies were conducted in this regard which showed some improvement in the severity of colitis following the dietary supplementation [37].

Although overall gut microbial richness and abundance did not change significantly from the baseline to the end of the trial in patients randomized to the two dietary groups, we found several specific bacterial taxa that changed significantly from the baseline to the end of the study in the two diet groups. Whilst we indicated that the CFG was associated with decreased *Bifidobacteriaceae, Lachnospiraceae*, *Clostridiaceae*, and *Ruminococcaceae*, the AID increased the abundance of *Bifidobacteriaceae, Lachnospiraceae*, and *Ruminococcaceae*. Furthermore, a significant relationship between changes in FCP levels and changes in the relative abundance of some bacteria was found in the present study. 

Alterations in gut microbiota and their related metabolites (e.g., bile acids, short-chain fatty acids, and tryptophan metabolites) have profound effects on immune maturation, immune homoeostasis, host energy metabolism, and the maintenance of mucosal integrity in IBD [38]. Furthermore, the association between dietary factors and gut microbial composition in UC patients has also been shown recently [39]. In a recent prospective cohort study, Godny et al. [40] reported that a reduction in fruit consumption was associated with shifts in the fecal microbiota that further correlated with the development of pouchitis in UC patients who underwent proctocolectomy. Although it has been suggested that the modulation of gut microbiota through dietary manipulations may result in favorable outcomes in IBD patients, the impact of dietary interventions on gut microbial composition in IBD patients has been evaluated in only a few RCTs [41]. A recent high-quality study demonstrated that diet accounted for a small proportion (3%) of the taxonomic variation between subjects and 0.7% of taxonomic variation longitudinally in IBD patients [42]. In another recent well-designed cross-over trial study on 17 UC patients in clinical remission, Fritsch et al. [43] reported a significant increase in *Bacteroidetes* and *Faecalibacterium prausnitzii* following a low-fat, high-fiber diet. In the present study, increased *Faecalibacterium* and *Blautia*, both of which belong to the phylum Firmicutes, were significantly associated with decreased FCP levels. This highlights the anti-inflammatory role these bacteria may have in our UC patients. A recent meta-analysis of 16 human studies on 1669 patients with CD or UC found a negative association between disease activity in IBD patients and the abundance of *Faecalibacterium prausnitzii* [44]. *Faecalibacterium prausnitzii* is a butyrate producer with several anti-inflammatory properties and an important bacterial contributor to intestinal homeostasis. Interestingly, we also found a significant inverse relationship between the DII or FCP and *Blautia* levels. *Blautia* belongs to the family Lachnospiraceae, which is known to exhibit anti-inflammatory properties by producing short-chain fatty acids. Its reduced abundance was shown previously in IBD patients [38]. These findings indicate that the increased intake of anti-inflammatory type foods (decreased DII score) could decrease colonic inflammation (i.e., decrease FCP) through significant increases in *Blautia*. However, the observed reduction in *Blautia* in the control group may be the result of increased inflammation. This needs to be investigated in future studies. 

Metabolomics, defined as the comprehensive study of all metabolites in biological samples, has been shown to have both diagnostic [21,45] and prognostic [46,47] potentials in different IBD settings. In addition, metabolomic approaches have been used recently to elucidate the mechanisms of response to different therapeutic interventions in IBD patients [48,49]. Metabolomics allows one to acquire a comprehensive picture of changes to metabolism, physiology, and cellular pathways, as well as to environmental factors such as diet or microbial alterations. 

In the present study, we found significant changes in the urinary, serum, and stool metabolomes of patients randomized to the AID following the 6-month trial. These changes in the metabolome suggest an important role for diet in altering host and microbial metabolism that may ultimately reduce colonic inflammation. 

Here, we discuss some of the more significantly changed metabolites and their relevance to our findings and to the dietary interventions. Urinary PC ae C38:3, which is a glycerophospholipid associated with low-calorie dieting [50], decreased significantly from the baseline to the end of the trial in our study. Furthermore, patients in the AID group had a significant decrease in their fecal acetone (a product of acetoacetate metabolism) following the dietary intervention. We had previously shown that higher serum acetone levels at the baseline were predictive of disease relapse in UC patients who were in clinical remission at the baseline^32^. Furthermore, a recent study on diarrhea-predominant irritable bowel syndrome patients indicated a significant decrease in the serum acetone level following 4 weeks of a synbiotic yogurt (*L. plantarum, L. fermentum*, and xylooligosaccharide) intervention [51]. The authors concluded that the decrease in acetone could be due to reduced inflammation associated with an increase in the *Lactobacilli* population. In our study, decreased colonic inflammation, as well as several changes in bacterial ASVs could play a role in the reduction in the acetone levels in these patients. 

We also found a significant decrease in xanthine levels in stool samples following the AID intervention. Xanthine is a purine base and an intermediate in the degradation of adenosine monophosphate to uric acid. It has been shown that bioactive compounds such as flavonoids that have antioxidant properties and have plant-based sources can decrease xanthine levels [52]. Interestingly, we also indicated that the decrease in xanthine level was associated with the decreased abundance of *Blautia*. Therefore, we speculate that the decreased xanthine levels in the AID group could be due to the increased intake of flavonoids by these participants, which may contribute to decreased colonic inflammation through significant changes in gut bacterial composition. 

We also found a significant increase in serum pyruvic acid (a tricarboxylic acid cycle metabolite related to energy metabolism), stool taurine, and urinary p-hydroxybenzoic acid. Taurine is a conditionally essential amino acid that plays an important role in many physiological functions in the human body. However, humans have a limited ability to synthesize taurine and are probably dependent in part on dietary taurine, which is found exclusively in foods of animal origin, including fish [53]. The observed increase in taurine levels in our study may be attributed to increased seafood intake in the AID group. P-hydroxybenzoic acid is an organic acid and a phenolic derivative of benzoic acid. Significant amounts of benzoic acid have been found in most berries. In addition, benzoic acid is a by-product of phenylalanine metabolism in bacteria and is produced when gut bacteria process polyphenols from plant sources [54]. Elevated urinary hydroxybenzoic acids have also been related to cocoa and tea intake [55]. Therefore, the observed changes in these metabolites can likely be attributed to the food contents of AID. However, the determination of their mechanistic roles in decreasing inflammation in UC requires further investigations. 

In our study, we have also found an increase in urinary carnosine levels following the AID. Carnosine (β-alanyl-L-histidine) is a histidine-containing dipeptide and has several biological roles, such as pH buffering, calcium regulation, anti-glycation, and antioxidant activity [56]. Although carnosine levels in urine have been suggested to be a biomarker of meat intake in healthy individuals [57], a recent randomized trial study showed that carnosine homeostasis was unaffected by a 6-month vegetarian diet [58]. Furthermore, in another study elevated urinary carnosine levels were related to higher adherence to the Mediterranean diet, while the authors found no relationship between carnosine and meat intake [59]. The authors suggested that the increase in carnosine levels could be due to other dietary factors related to the Mediterranean diet, such as higher vitamin B6 intake. 

It should be noted that in the present study, we also used an integrative approach to investigate the mechanisms by which following the anti-inflammatory diet could prevent increases of colonic inflammation in UC patients. We found that the potential benefits of increasing the intake of anti-inflammatory foods (as shown by a decreased DII score) are modulated by the direct and indirect effects of the diet on gut bacterial composition and several host- and bacterial-related metabolites in stool, urine, and serum. In this regard, we found a statistically significant correlation between increases in FCP and increased levels of trimethylamine in the stool samples from the baseline to the study endpoint. Trimethylamine is a precursor of trimethylamine N-oxide (TMAO) that is formed from dietary phosphatidylcholine and carnitine via microbiota-dependent pathways in the gut. Increased fecal TMAO has been shown in UC patients [60]. Furthermore, it has been reported that CD patients had higher levels of trimethylamine in their stools, which decreased significantly following exclusive enteral nutrition [61]. These findings emphasize the potential role of trimethylamine in IBD, which requires further investigations. 

Our study is among the first RCTs investigating the potential benefits of a dietary intervention for the maintenance of remission in UC patients. The AID as a set of dietary recommendations was designed to be followed by individuals for 6 months by giving them the opportunity to select different food items based on their personal dietary preferences. Although an IBD specifically designed catered diet (low fat, high fiber diet [43]) for 4 weeks has recently been shown to improve inflammation in UC patients, its high costs (~20,000 USD/year) and feasibility issues advocates for the designing of dietary recommendations and menus that are easy to follow for patients in free-living settings. 

Our study has a few methodological limitations that require further discussion. The relatively small sample size is a main limiting factor in the current study and this likely contributed to our inability to reach statistical significance for the clinical relapse rate (the primary outcome of our study) between the two diet groups. However, we did detect a significant role of the AID in the modest decrease and the prevention of the increase in FCP, an objective marker for subclinical intestinal inflammation and prognosis for future relapses. In comparison to the control diet, this finding is encouraging, and it suggests that performing a larger study to evaluate the contribution of the AID or other types of dietary manipulation (e.g., the Mediterranean diet) for the prevention of disease relapse in UC patients. Furthermore, the assessment of dietary intake changes from the baseline to the end of the trial was based on self-reported 24 h dietary recalls, which are subject to recall bias and inaccurate reporting. However, we tried to attenuate this issue by using frequent validated 24 h dietary recalls and assessment of the changes in metabolites as objective measures of the dietary intake. Indeed, the results from our metabolomics analysis confirm the compliance of the patients with their assigned diet.

In conclusion, we have shown that adherence to an anti-inflammatory diet can prevent subclinical colonic inflammation in UC patients who are in clinical remission. This finding was accompanied by significant changes in the metabolomic and gut microbial profiles of AID subjects. These results are promising and should encourage the future development of well-designed RCTs with larger sample sizes to further assess dietary interventions for the maintenance of remission in UC patients. 

## Figures and Tables

**Table 1:** Demographic and clinical characteristics of study participants at baseline.

Characteristics		AID (*n* = 26)	CFG (*n* = 27)	*p*-Value
Age, years		36.5 (30.0–55.5)	43.0 (25.0–54.0)	0.64
Females, n (%)		15 (57.7)	19 (70.4)	0.34
Current smoker, n (%)		1 (3.8)	1 (3.7)	1.00
University degree, n (%)		16 (61.5)	10 (37.0)	0.07
Body mass index, kg/m^2^		25.2 (22.1–29.2)	24.2 (22.6–27.6)	0.78
Partial Mayo score		0 (0–0)	0 (0–0)	1.00
Years since diagnosis, years		9.0 (5.5–12.8)	6.0 (3.0–13.0)	0.35
Duration of remission, months		6.0 (3.0–9.5)	6.0 (4.0–8.0)	0.96
UC subtype, n (%)	Proctitis Left-sided colitis Pancolitis	3 (11.5) 12 (46.2) 11 (42.3)	3 (11.1) 10 (37.0) 14 (51.9)	0.77
UC medications, n (%)	No UC medication 5-aminosalicylic acid Immunosuppressants Biologics (Anti-TNF)	2 (7.7) 18 (69.2) 9 (34.6) 7 (26.9)	3 (11.1) 22 (81.5) 7 (25.9) 6 (22.2)	0.67 0.30 0.49 0.69
C-reactive protein, mg/L		1.1 (0.7–2.0)	1.2 (0.5–3.7)	0.67
Fecal calprotectin, µg/g		129 (70–266)	184 (85–483)	0.43
Fecal calprotectin < 150 µg/g, n (%)		16 (61.5)	13 (48.1)	0.41
Short Inflammatory Bowel Disease Questionnaire		5.5 (4.9–6.4)	5.0 (5.6–6.0)	0.99

**Table 2:** Concentration of major metabolites in different biofluids responsible for the discrimination of metabolome from baseline to month 6 or time of relapse in the anti-inflammatory diet group.

Metabolites (Biofluid/Secreta)	Time		*p*-Value ^1^	VIP Score
	Baseline	Month6/Relapse		
PC ae C38:3 (urine), µM/mM creatinine	0.0012 (0.0009–0.0022)	0.0008 (0.0004–0.0013)	0.003	2.20
PC ae C38:5 (urine), µM/mM creatinine	0.0002 (0.0001–0.0006)	0.0006 (0.0002–0.0016)	0.03	1.65
Acetone (stool), µM/g	0.0975 (0.0422–0.2875)	0.0440 (0.0320–0.1387)	0.021	1.62
Carnosine (urine), µM/mM creatinine	0.4760 (0.2304–1.6037)	1.1171 (0.3931–2.6940)	0.026	1.47
Pyruvic acid (serum), µM	34.2000 (23.3750–50.7000)	45.4000 (35.0750–62.5250)	0.007	1.35
Taurine (stool), µM/g	0.2390 (0.1140–1.7060)	0.7565 (0.1623–2.3445)	0.049	1.26
p-Hydroxybenzoic acid (urine), µM/mM creatinine	0.3433 (0.2136–0.7777)	0.7265 (0.3568–1.6008)	0.012	1.15
Xanthine (stool), µM/g	0.0945 (0.0630–0.1358)	0.0725 (0.0438–0.1215)	0.025	1.13

## Data Availability

Dataset will be shared upon request to the corresponding author.

## Supplementary Materials

The following supporting information can be downloaded at: https://www.mdpi.com/article/10.3390/nu14163294/s1, Figure S1: gut bacterial composition (family level) in stool samples collected from patients randomized to the anti-inflammatory diet (AID) and Canada’s Food Guide (CFG) groups at baseline (0) and at month 6 or time of relapse (6), Figure S2: comparison of alpha diversity scores from baseline to month 6 or time of relapse in patients randomized to the anti-inflammatory diet (AID) and Canada’s Food Guide (CFG) groups showed no significant changes in either Chao1 (A) estimator or Shannon index (B), Table S1: comparison of changes in dietary intake of foods and nutrients from baseline to month 6 between the two diet groups.

## References

[1] Ungaro R., Mehandru S., Allen P.B., Peyrin-Biroulet L., Colombel J.F. (2017). Ulcerative colitis. Lancet.

[2] Ramos G.P., Papadakis K.A. (2019). Mechanisms of Disease: Inflammatory Bowel Diseases. Mayo Clin. Proc..

[3] Keshteli A.H., Madsen K.L., Dieleman L. (2019). Diet in the Pathogenesis and Management of Ulcerative Colitis; A Review of Randomized Controlled Dietary Interventions. Nutrients.

[4] Khalili H., Chan S.S.M., Lochhead P., Ananthakrishnan A.N., Hart A.R., Chan A.T. (2018). The role of diet in the aetiopathogenesis of inflammatory bowel disease. Nat. Rev. Gastroenterol. Hepatol..

[5] Reddavide R., Rotolo O., Caruso M.G., Stasi E., Notarnicola M., Miraglia C., Nouvenne A., Meschi T., Angelis G.L.D., Di Mario F. (2018). The role of diet in the prevention and treatment of Inflammatory Bowel Diseases. Acta Biomed..

[6] Ananthakrishnan A.N., Bernstein C.N., Iliopoulos D., Macpherson A., Neurath M.F., Ali R.A.R., Vavricka S.R., Fiocchi C. (2018). Environmental triggers in IBD: A review of progress and evidence. Nat. Rev. Gastroenterol. Hepatol..

[7] Levine A., Boneh R.S., Wine E. (2018). Evolving role of diet in the pathogenesis and treatment of inflammatory bowel diseases. Gut.

[8] Casellas F., Arenas J.I., Baudet J.S., Fábregas S., García N., Gelabert J., Medina C., Ochotorena I., Papo M., Rodrigo L. (2005). Impairment of Health-Related Quality of Life in Patients with Inflammatory Bowel Disease: A Spanish Multicenter Study. Inflamm. Bowel Dis..

[9] Choi C.-H.R., Al Bakir I., Hart A.L., Graham T.A. (2017). Clonal evolution of colorectal cancer in IBD. Nat. Rev. Gastroenterol. Hepatol..

[10] Vedamurthy A., Ananthakrishnan A.N. (2019). Influence of Environmental Factors in the Development and Outcomes of Inflammatory Bowel Disease. Gastroenterol. Hepatol..

[11] Martin T.D., Chan S.S.M., Hart A.R. (2015). Environmental Factors in the Relapse and Recurrence of Inflammatory Bowel Disease: A Review of the Literature. Am. J. Dig. Dis..

[12] Limketkai B.N., Iheozor-Ejiofor Z., Gjuladin-Hellon T., Parian A., Matarese L.E., Bracewell K., Macdonald J.K., Gordon M., Mullin G.E. (2019). Dietary interventions for induction and maintenance of remission in inflammatory bowel disease. Cochrane Database Syst. Rev..

[13] De Vos M., Louis E.J., Jahnsen J., Vandervoort J.G., Noman M., Dewit O., D’haens G.R., Franchimont D., Baert F.J., Torp R.A. (2013). Consecutive fecal calprotectin measurements to predict relapse in patients with ulcerative colitis receiving infliximab maintenance therapy. Inflamm. Bowel Dis..

[14] Harris P.A., Taylor R., Thielke R., Payne J., Gonzalez N., Conde J.G. (2009). Research electronic data capture (REDCap)—A metadata-driven methodology and workflow process for providing translational research informatics support. J. Biomed. Inform..

[15] Health Canada (2007). Eating Well with Canada’s Food Guide.

[16] Soria-Contreras D.C., Bell R.C., McCargar L.J., Chan C.B. (2014). Feasibility and Efficacy of Menu Planning Combined with Individual Counselling to Improve Health Outcomes and Dietary Adherence in People with Type 2 Diabetes: A Pilot Study. Can. J. Diabetes.

[17] Irvine E.J., Zhou Q., Thompson A.K. (1996). The Short Inflammatory Bowel Disease Questionnaire: A quality of life instrument for community physicians managing inflammatory bowel disease. CCRPT Investigators. Canadian Crohn’s Relapse Prevention Trial. Am. J. Gastroenterol..

[18] National Cancer Institute Automated Self-Administered 24-Hour (ASA24®) Dietary Assessment Tool. https://epi.grants.cancer.gov/asa24/..

[19] Shivappa N., Steck S.E., Hurley T.G., Hussey J.R., Hébert J.R. (2014). Designing and developing a literature-derived, population-based dietary inflammatory index. Public Health Nutr..

[20] Bouatra S., Aziat F., Mandal R., Guo A.C., Wilson M.R., Knox C., Bjorndahl T.C., Krishnamurthy R., Saleem F., Liu P. (2013). The Human Urine Metabolome. PLoS ONE.

[21] Keshteli A.H., Madsen K.L., Mandal R., Boeckxstaens G.E., Bercik P., De Palma G., Reed D.E., Wishart D., Vanner S., Dieleman L.A. (2019). Comparison of the metabolomic profiles of irritable bowel syndrome patients with ulcerative colitis patients and healthy controls: New insights into pathophysiology and potential biomarkers. Aliment. Pharmacol. Ther..

[22] Psychogios N., Hau D.D., Peng J., Guo A.C., Mandal R., Bouatra S., Sinelnikov I., Krishnamurthy R., Eisner R., Gautam B. (2011). The Human Serum Metabolome. PLoS ONE.

[23] Zordoky B., Sung M.M., Ezekowitz J., Mandal R., Han B., Bjorndahl T.C., Bouatra S., Anderson T., Oudit G.Y., Wishart D.S. (2015). Metabolomic Fingerprint of Heart Failure with Preserved Ejection Fraction. PLoS ONE.

[24] Callahan B.J., Mcmurdie P.J., Rosen M.J., Han A.W., Johnson A.J.A., Holmes S.P. (2016). DADA2: High-resolution sample inference from Illumina amplicon data. Nat. Methods.

[25] Chong J., Soufan O., Li C., Caraus I., Li S., Bourque G., Wishart D.S., Xia J. (2018). MetaboAnalyst 4.0: Towards more transparent and integrative metabolomics analysis. Nucleic Acids Res..

[26] McMurdie P.J., Holmes S. (2013). phyloseq: An R package for reproducible interactive analysis and graphics of microbiome census data. PLoS ONE.

[27] Basu S., Duren W., Evans C.R., Burant C.F., Michailidis G., Karnovsky A. (2017). Sparse network modeling and metscape-based visualization methods for the analysis of large-scale metabolomics data. Bioinformatics.

[28] Shannon P., Markiel A., Ozier O., Baliga N.S., Wang J.T., Ramage D., Amin N., Schwikowski B., Ideker T. (2003). Cytoscape: A software environment for integrated models of Biomolecular Interaction Networks. Genome Res..

[29] Rizzello F., Spisni E., Giovanardi E., Imbesi V., Salice M., Alvisi P., Valerii M.C., Gionchetti P. (2019). Implications of the Westernized Diet in the Onset and Progression of IBD. Nutrients.

[30] Jowett S.L., Seal C.J., Pearce M.S., Phillips E., Gregory W., Barton J.R., Welfare M.R. (2004). Influence of dietary factors on the clinical course of ulcerative colitis: A prospective cohort study. Gut.

[31] Limdi J.K., Aggarwal D., McLaughlin J.T. (2016). Dietary Practices and Beliefs in Patients with Inflammatory Bowel Disease. Inflamm. Bowel Dis..

[32] Keshteli A.H., Brand F.F.V.D., Madsen K.L., Mandal R., Valcheva R., I Kroeker K., Han B., Bell R.C., Cole J., Hoevers T. (2017). Dietary and metabolomic determinants of relapse in ulcerative colitis patients: A pilot prospective cohort study. World J. Gastroenterol..

[33] Li F., Liu X., Wang W., Zhang D. (2015). Consumption of vegetables and fruit and the risk of inflammatory bowel disease: A meta-analysis. Eur. J. Gastroenterol. Hepatol..

[34] Mozaffari H., Daneshzad E., Larijani B., Bellissimo N., Azadbakht L. (2020). Dietary intake of fish, n-3 polyunsaturated fatty acids, and risk of inflammatory bowel disease: A systematic review and meta-analysis of observational studies. Eur. J. Nutr..

[35] Shivappa N., Hébert J.R., Rashvand S., Rashidkhani B., Hekmatdoost A. (2016). Inflammatory Potential of Diet and Risk of Ulcerative Colitis in a Case-Control Study from Iran. Nutr. Cancer.

[36] Scaioli E., Liverani E., Belluzzi A. (2017). The Imbalance between n-6/n-3 Polyunsaturated Fatty Acids and Inflammatory Bowel Disease: A Comprehensive Review and Future Therapeutic Perspectives. Int. J. Mol. Sci..

[37] Weisshof R., Chermesh I. (2015). Micronutrient deficiencies in inflammatory bowel disease. Curr. Opin. Clin. Nutr. Metab. Care.

[38] Zhuang X., Liu C., Zhan S., Tian Z., Li N., Mao R., Zeng Z., Chen M. (2021). Gut Microbiota Profile in Pediatric Patients with Inflammatory Bowel Disease: A Systematic Review. Front. Pediatr..

[39] Weng Y.J., Gan H.Y., Li X., Huang Y., Li Z.C., Deng H.M., Chen S.Z., Zhou Y., Wang L.S., Han Y.P. (2019). Correlation of diet, microbiota and metabolite networks in inflammatory bowel disease. J. Dig. Dis..

[40] Godny L., Maharshak N., Reshef L., Goren I., Yahav L., Fliss-Isakov N., Gophna U., Tulchinsky H., Dotan I. (2019). Fruit Consumption is Associated with Alterations in Microbial Composition and Lower Rates of Pouchitis. J. Crohn’s Colitis.

[41] McIlroy J., Ianiro G., Mukhopadhya I., Hansen R., Hold G.L. (2018). Review article: The gut microbiome in inflammatory bowel disease-avenues for microbial management. Aliment. Pharmacol. Ther..

[42] Lloyd-Price J., Arze C., Ananthakrishnan A.N., Schirmer M., Avila-Pacheco J., Poon T.W., Andrews E., Ajami N.J., Bonham K.S., Brislawn C.J. (2019). Multi-omics of the gut microbial ecosystem in inflammatory bowel diseases. Nature.

[43] Fritsch J., Garces L., Quintero M.A., Pignac-Kobinger J., Santander A.M., Fernández I., Ban Y.J., Kwon D., Phillips M.C., Knight K. (2021). Low-Fat, High-Fiber Diet Reduces Markers of Inflammation and Dysbiosis and Improves Quality of Life in Patients With Ulcerative Colitis. Clin. Gastroenterol. Hepatol..

[44] Zhao H., Xu H., Chen S., He J., Zhou Y., Nie Y. (2021). Systematic review and meta-analysis of the role of *Faecalibacterium prausnitzii* alteration in inflammatory bowel disease. J. Gastroenterol. Hepatol..

[45] De Preter V. (2015). Metabolomics in the Clinical Diagnosis of Inflammatory Bowel Disease. Dig. Dis..

[46] Probert F., Walsh A., Jagielowicz M., Yeo T., Claridge T.D.W., Simmons A., Travis S., Anthony D.C. (2018). Plasma Nuclear Magnetic Resonance Metabolomics Discriminates Between High and Low Endoscopic Activity and Predicts Progression in a Prospective Cohort of Patients With Ulcerative Colitis. J. Crohns Colitis.

[47] Keshteli A.H., Tso R., Dieleman L.A., Park H., Kroeker K.I., Jovel J., Gillevet P.M., Sikaroodi M., Mandal R., Fedorak R.N. (2018). A Distinctive Urinary Metabolomic Fingerprint Is Linked With Endoscopic Postoperative Disease Recurrence in Crohn’s Disease Patients. Inflamm. Bowel Dis..

[48] Nakanishi M., Matz A., Klemashevich C., Rosenberg D.W. (2019). Dietary Walnut Supplementation Alters Mucosal Metabolite Profiles During DSS-Induced Colonic Ulceration. Nutrients.

[49] Paramsothy S., Nielsen S., Kamm M.A., Deshpande N.P., Faith J.J., Clemente J.C., Paramsothy R., Walsh A.J., van den Bogaerde J., Samuel D. (2019). Specific Bacteria and Metabolites Associated with Response to Fecal Microbiota Transplantation in Patients with Ulcerative Colitis. Gastroenterology.

[50] Menni C., Zhai G., MacGregor A., Prehn C., Römisch-Margl W., Suhre K., Adamski J., Cassidy A., Illig T., Spector T.D. (2013). Targeted metabolomics profiles are strongly correlated with nutritional patterns in women. Metabolomics.

[51] Noorbakhsh H., Yavarmanesh M., Mortazavi S.A., Adibi P., Moazzami A.A. (2019). Metabolomics analysis revealed metabolic changes in patients with diarrhea-predominant irritable bowel syndrome and metabolic responses to a synbiotic yogurt intervention. Eur. J. Nutr..

[52] Jiménez-Girón A., Ibáñez C., Cifuentes A., Simó C., Muñoz-González I., Martín-Álvarez P.J., Bartolomé B., Moreno-Arribas M.V. (2015). Faecal Metabolomic Fingerprint after Moderate Consumption of Red Wine by Healthy Subjects. J. Proteome Res..

[53] Lambert I.H., Kristensen D.M., Holm J.B., Mortensen O.H. (2015). Physiological role of taurine—From organism to organelle. Acta Physiol..

[54] Williamson G., Clifford M.N. (2010). Colonic metabolites of berry polyphenols: The missing link to biological activity?. Br. J. Nutr..

[55] Medina S., Domínguez-Perles R., Ferreres F., Tomás-Barberán F.A., Gil-Izquierdo Á. (2013). The effects of the intake of plant foods on the human metabolome. TrAC Trends Anal. Chem..

[56] Boldyrev A.A., Aldini G., Derave W. (2013). Physiology and Pathophysiology of Carnosine. Physiol. Rev..

[57] Cheung W., Keski-Rahkonen P., Assi N., Ferrari P., Freisling H., Rinaldi S., Slimani N., Zamora-Ros R., Rundle M., Frost G. (2017). A metabolomic study of biomarkers of meat and fish intake. Am. J. Clin. Nutr..

[58] Blancquaert L., Baguet A., Bex T., Volkaert A., Everaert I., Delanghe J., Petrovic M., Vervaet C., De Henauw S., Constantin-Teodosiu D. (2018). Changing to a vegetarian diet reduces the body creatine pool in omnivorous women, but appears not to affect carnitine and carnosine homeostasis: A randomised trial. Br. J. Nutr..

[59] Almanza-Aguilera E., Urpi-Sarda M., Llorach R., Vázquez-Fresno R., Garcia-Aloy M., Carmona F., Sanchez A., Madrid-Gambin F., Estruch R., Corella D. (2017). Microbial metabolites are associated with a high adherence to a Mediterranean dietary pattern using a 1H-NMR-based untargeted metabolomics approach. J. Nutr. Biochem..

[60] Santoru M.L., Piras C., Murgia A., Palmas V., Camboni T., Liggi S., Ibba I., Lai M.A., Orrù S., Blois S. (2017). Cross sectional evaluation of the gut-microbiome metabolome axis in an Italian cohort of IBD patients. Sci. Rep..

[61] Diederen K., Li J.V., Donachie G.E., De Meij T.G., De Waart D.R., Hakvoort T.B.M., Kindermann A., Wagner J., Auyeung V., Velde A.A.T. (2020). Exclusive enteral nutrition mediates gut microbial and metabolic changes that are associated with remission in children with Crohn’s disease. Sci. Rep..

