# Supplementary material for: Anti-Inflammatory Diet Prevents Subclinical Colonic Inflammation and Alters Metabolomic Profile of Ulcerative Colitis Patients in Clinical Remission
Source: Nutrients. 2022 Aug 11;14(16):3294. doi: 10.3390/nu14163294 (PMC9414437; doi:10.3390/nu14163294)
Supplement: Supplementary file 1 [file nutrients-14-03294-s001.zip › nutrients-1864412-supplementary.pdf]

Table S1. Comparison of changes in dietary intake of foods and nutrients from baseline to month 6 between the two diet groups.

	AID		P-value[1]	CFG		P-value[2]	P-value[3]
	**Baseline**	Month 1-6		Baseline	Month 1-6		
Energy (kcal/d)	1882.4±524.7	1958.9±551.9	0.29	1950.2±616.4	1911.5±568.5	0.45	0.26
Protein (%E)	16.2±4.3	17.3±3.3	0.32	19.9±5.7	18.7±4.4	0.11	0.21
Carbohydrate (%E)	49.4±8.4	49.8±8.9	0.78	47.1±11.5	45.3±9.3	0.30	0.31
Fat (%E)	35.8±9.6	33.4±6.8	0.15	33.5±7.5	34.5±8.3	0.37	0.21
Fiber (g/d)	20.2±6.6	22.8±6.7	0.04	22.5±11.8	22.3±8.3	0.61	0.09
Vitamin A (µg/d)	1018.1±794.1	1134.9±731.7	0.13	1198.5±696.9	900.7±440.2	0.03	0.01
Niacin (mg/d)	24.8±17.5	27.2±14.7	0.23	29.2±10.0	27.8±11.1	0.27	0.06
Zinc (mg/d)	10.6±5.3	11.7±6.1	0.05	17.7±11.7	16.1±8.0	0.45	0.00
Phosphorus (mg/d)	1408.6±626.6	1561.4±548.8	0.05	1571.9±637.3	1472.5±435.0	0.36	0.02
Selenium (µg/d)	110.4±61.8	122.2±39.6	0.04	128.4±36.6	121.6±25.7	0.34	0.01
Choline (mg/d)	297.8±130.8	334.5±118.3	0.07	367.6±176.3	332.1±132.3	0.46	0.05
Seafood (ounce equivalents/d)	0.7±1.2	1.4±1.5	0.01	1.2±2.1	1.1±1.3	0.82	0.07
Yogurt (cup equivalents/d)	0.3±0.3	0.5±0.3	<0.01	0.1±0.2	0.2±0.2	0.28	0.06

[1] for comparison of baseline vs. month 6/relapse levels in the anti-inflammatory diet (AID) group; [2] for comparison of baseline vs. month 6/relapse levels in the Canada's Food Guide (CFG) group; [3] for comparison of changes from baseline to month 6/relapse between AID and CFG groups.

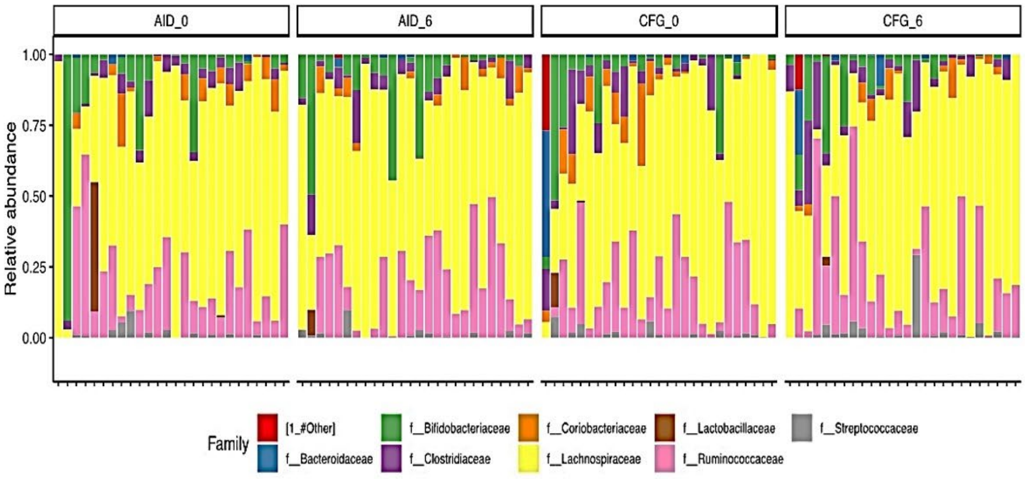

Figure S1. Gut bacterial composition (family level) in stool samples collected from patients randomized to the anti-inflammatory diet (AID) and Canada's Food Guide (CFG) groups at baseline (0) and at month 6 or time of relapse (6).

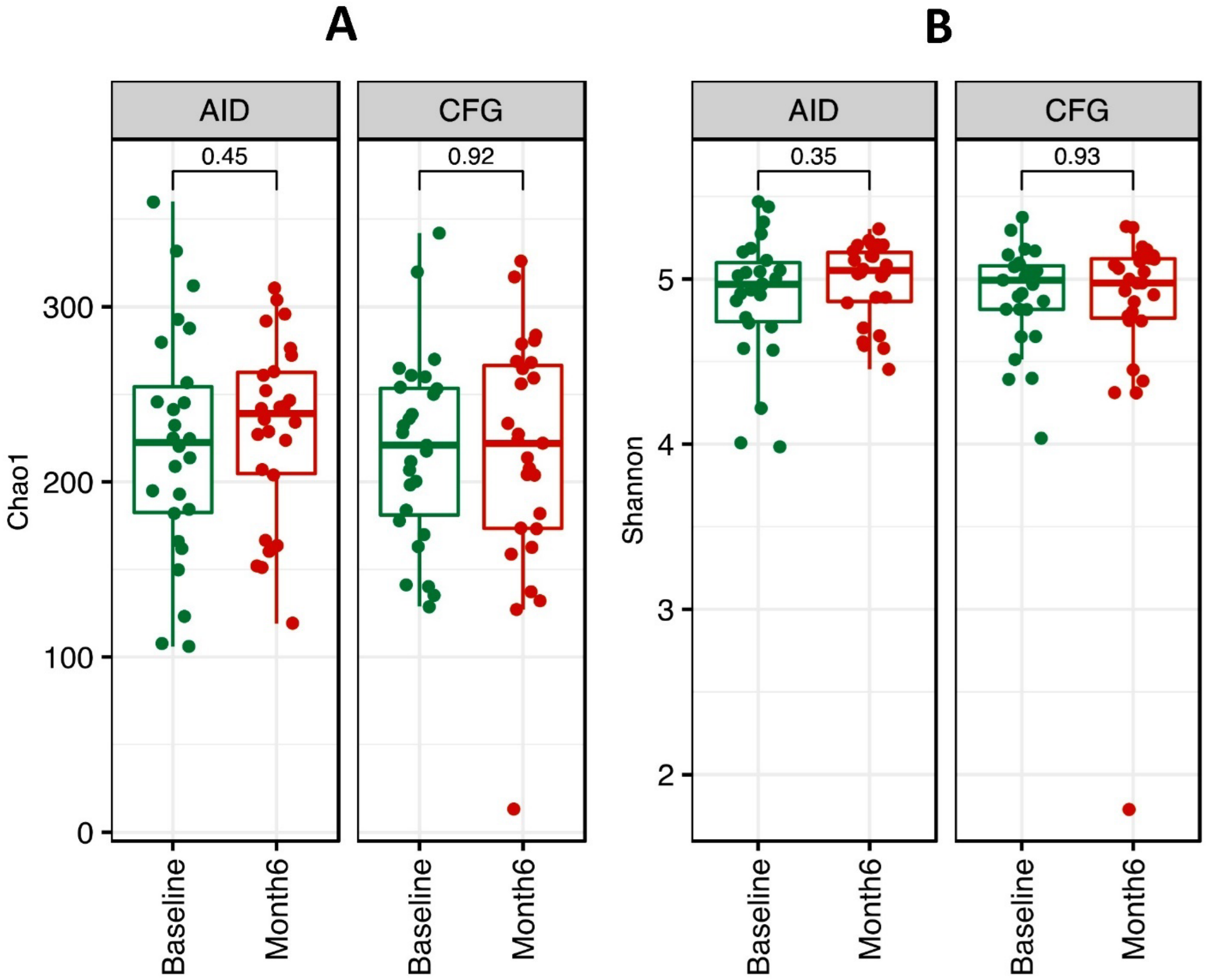

Figure S2. Comparison of alpha diversity scores from baseline to month 6 or time of relapse in patients randomized to the anti-inflammatory diet (AID) and Canada's Food Guide (CFG) groups showed no significant changes in either Chao1 (**A**) estimator or Shannon index (**B**).
